# Validity of tools used for surveying physicians about their interactions with pharmaceutical company: a systematic review

**DOI:** 10.1186/s13104-015-1709-4

**Published:** 2015-11-25

**Authors:** Tamara Lotfi, Rami Z. Morsi, Nada Zmeter, Mohammad W. Godah, Lina Alkhaled, Lara A. Kahale, Hala Nass, Hneine Brax, Racha Fadlallah, Elie A. Akl

**Affiliations:** Department of Clinical Research Institute, American University of Beirut, Beirut, Lebanon; Department of Pediatrics and Adolescent Medicine, Faculty of Medicine, American University of Beirut, Beirut, Lebanon; Faculty of Health Sciences, American University of Beirut, Beirut, Lebanon; Faculty of Medicine, University of Damascus, Damascus, Syria; Faculty of Medicine, Université Saint Joseph, Beirut, Lebanon; Department of Internal Medicine, American University of Beirut, Riad-El-Solh Beirut 1107 2020, P.O. Box: 11-0236, Beirut, Lebanon; Department of Clinical Epidemiology and Biostatistics, McMaster University, Hamilton, ON Canada

## Abstract

**Background:**

There is evidence that physicians’ prescription behavior is negatively affected by the extent of their interactions with pharmaceutical companies. In order to develop and implement policies and interventions for better management of interactions, we need to understand physicians’ perspectives on this issue. Surveys addressing physicians’ interactions with pharmaceutical companies need to use validated tools to ensure the validity of their findings.

**Objective:**

To assess the validity of tools used in surveys of physicians about the extent and nature of their interactions with pharmaceutical companies, and about their knowledge, beliefs and attitudes towards such interactions; and to identify those tools that have been formally validated.

**Methods:**

We developed a search strategy with the assistance of a medical librarian. We electronically searched MEDLINE and EMBASE databases in September 2015. Teams of two reviewers conducted data selection and data abstraction. They identified eligible studies in one table and then abstracted the relevant data from the studies with validated tools in another table. Tables were piloted and standardized.

**Results:**

We identified one validated questionnaire out of the 11 assessing the nature and extent of the interaction, and three validated questionnaires out of the 47 assessing knowledge, beliefs and attitudes of physicians toward the interaction. None of these validated questionnaires were used in more than one survey.

**Conclusion:**

The available supporting evidence of the issue of physicians’ interaction with pharmaceutical company is of low quality. There is a need for research to develop and validate tools to survey physicians about their interactions with pharmaceutical companies.

## Background

Social, economic and public-health sectors are interested in the elements of poor prescription practices [1, 2]. There is evidence that physicians’ prescription behavior is negatively affected by the extent of their interactions with pharmaceutical companies [3, 4].

A large number of qualitative and quantitative studies aimed to understand physicians’ knowledge, beliefs and attitudes towards their interaction with pharmaceutical company representatives. In at least two studies, physicians have denied being influenced by pharmaceutical promotion while claiming it influenced their colleagues [5, 6].

There have been many initiatives to manage the financial relationships between industry and physicians. For example, the Institute of Medicine published in 2009 the “Conflict of Interest in Medical Research, Education, and Practice” report which included recommendations to addressing those relationship [7]. More recently, the Physician Payment Sunshine Act has required pharmaceutical and medical device companies to publicly report payments to physicians and teaching hospitals, as well as certain ownership interests [8].

In order to develop and implement policies and interventions for better management of interactions [9], we need to understand their extent, as well as physicians’ perspectives towards interaction with pharmaceutical companies. Studies of physicians’ knowledge, attitudes and beliefs need to measure them using validated tools.

The study objectives were to:

1. Assess the validity of tools used in surveys of physicians about the extent and nature of their interactions with pharmaceutical companies, and about their knowledge, beliefs and attitudes of towards such interactions.
2. Identify and describe survey tools that have been formally validated

## Methods

### Eligibility criteria

The inclusion criteria for our first objective (assessing validity of tools used in surveys) were:

- Types of study design: quantitative survey studies
- Types of participants: Practicing physicians; we used no restrictions on country of practice;
- Types of interactions: any form of interaction between physicians and pharmaceutical companies or their representatives;
- Types of outcomes: extent and nature; knowledge, beliefs, attitude [10].

The exclusion criteria for our first objective are:

- Studies restricted to “residents” or “medical students”
- Studies not published in English.

The inclusion criteria for our second objective (identifying and describing validated tools) were:

- Tools used in one of the studies included under the first objective
- Tools assessed for criterion validity and/or construct validity (see Appendix 1 for definitions). We did not include tools assessed only for face and/or content validity given they represent perceptions and judgments of experts regarding the content of the tool [11].

### Search strategy

We developed a search strategy with the assistance of a medical librarian (Appendix 2). We electronically searched MEDLINE and EMBASE databases in September 2015. We did not apply any search filter. We also screened the references lists of included studies and the grey literature (e.g., theses). Last, we searched the Health and Psychosocial Instruments (HaPI) database to screen for indexed validated tools relevant to our study.

### Selection of studies

Teams of two reviewers screened the titles and abstracts of the identified studies in duplicate and independently. Then they used standardized forms to screen the full texts of the studies judged as potentially eligible by at least one of the reviewers. They compared results, and resolved disagreement by discussion. They sought the input of a third reviewer as needed.

### Data collection

Teams of two reviewers abstracted data from included studies. They compared results, and resolved disagreement by discussion. When needed they sought the input of a third reviewer. They used pilot tested standardized data abstraction forms.

- For each study included under objective 1, we noted whether the tool was newly developed (versus a previously developed, versus a modified version of a previously developed tool) and whether or not it has been validated.
- For each validated tool included under objective 2, we noted the concepts it measured (e.g., extent, nature, attitudes, beliefs, knowledge); how it was developed; and how it was validated.

### Data analysis and synthesis

We calculated the kappa statistic to assess the agreement between reviewers for full text screening. We summarized the findings in both narrative and tabular formats, as the nature of the data was not amenable to a meta-analysis. We reported the results separately for tools measuring extent and nature, and for tools measuring knowledge, beliefs and attitudes.

## Results

**Fig. 1:**
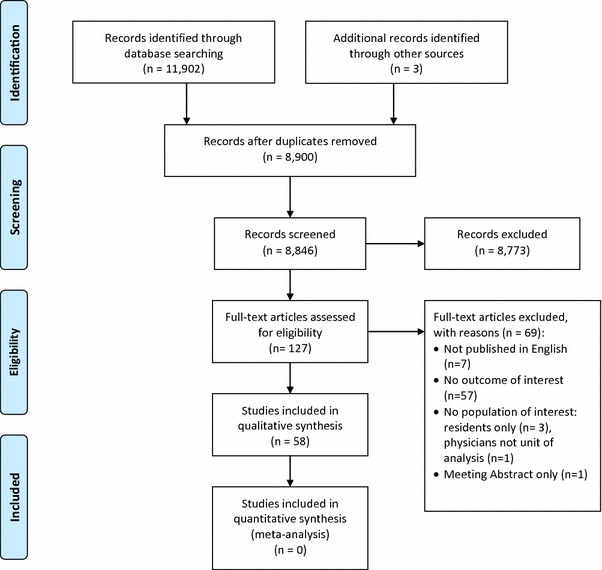
Study flow

Figure 1 shows the results of the search and of study selection. The kappa statistic for full-text screening was high at 0.893. We identified 11 eligible surveys assessing nature and extent of the interaction, and 47 eligible surveys assessing knowledge, beliefs and attitudes.

### Nature and extent of the interaction

#### Validity of tools used in surveys

Table 1 shows the 11 included studies and the validity of their tools. Nine studies reported developing a new tool [12–20], and two reported modifying a previously developed “validated” tool [21]. Of these 11 studies, only one used a tool that met our criteria for validity [19]. Of the remaining ten studies, three assessed content validity [18, 20, 21].

**Table 1:** Description of the originality and validity of tools used in surveys assessing the extent and nature of interaction between physicians and pharmaceutical companies

	Newly developed	Previously developed, modified
Validated	Madhavan [19]	
Non validated	Alosaimi [18] Halperin [12] Hemminki [13] Hossain [14] Lurie [32] Scheffer [16] Seidel [17] Venkateraman [33]	Alosaimi [21] Ferrari [20]

#### Validated survey tools

As stated above, only one tool assessing the nature and extent of the interaction fit our criteria for a ‘validated tool’ (Table 2). That tool measured the concept of “gift giving”. While the report did not provide details about the development of the tool, it described evaluating face validity as well as construct validity using principal component factor analysis of the attitudinal items.

**Table 2:** Detailed description of validated tools used for surveying physicians about their interactions with pharmaceutical company

Study ID	Concept(s) measured by instrument	Development methods	Validation methods
Madhavan [19]	Extent of giving	Not described	Face validity Assessment of theoretical and construct validity using principal component factor analysis of the attitudinal items
Andaleeb [22]	Attitudes toward pharmaceutical company representatives	Exploration of “secondary sources of information” In-depth interviews of physicians and pharmaceutical company representatives Development of a preliminary questionnaire Pre-testing on three physicians	Face and content validity Construct validity Factor analysis with correlation matrix suggesting discriminant validity
Fernandez [2]	Importance of sources of influence	Bibliographical review and assessment by experts Tested on a sample of 124 GPs	Content and construct validity Multiple correlation analysis and a factorial analysis
McKinney [23]	Attitudes of physicians toward interaction with pharmaceutical company representatives Perception of the value of a gift likely to compromise a physician’s judgment	Reviewed and edited by three individuals with expertise in questionnaire design, education, or pharmaceutical detailing	Construct validity Expert review and factor analysis

### Knowledge, beliefs and attitudes

#### Validity of survey tools

Table 3 categorizes the 47 included studies and the validity of their tools. Of these studies, 37 developed new tools, five used previously developed tools, and five modified previously developed tools. Of the 47 included studies, only three used tools that met our criteria for validity [2, 22, 23]. Of the remaining 40 studies, four studies evaluated content validity [20, 24–27] and one evaluated face validity [25].

**Table 3:** Description of the originality and validity of tools used in surveys assessing knowledge, beliefs and attitudes of physicians towards their interaction with pharmaceutical companies

	Newly developed	Previously developed, used as is	Previously developed, modified
Validated	Fernandez [2] McKinney [23] Andaleeb [22]		Ferrari [20]
Non validated	Mikhael [34] Indhumati [35] Tabas [36] Loh [27] Thomson [26] Skoglund [25] De Gara [37] Evans [38] Magzoub [39] Saito [40] Alghasham [41] Ellison [42] Ross [43] Burashnikova [44] Fortuna [45] Tobin [46] Morgan [6] Brett [47] Rutledge [48] De Las Cuevas [49] Spiller [50] Jones [51] Figueiras [1] Guldal [52] Gaedeke [53] Gibbons [54] Creyer [55] Gaither [56] Gaither [57] Banks 1992 [58] Hayes [59] Stross [60] Hull [61] Evans [62]	Siddiqi [63] Macneill [24] Alssageer [64] Stoddard [65] Sara [66]	Oshikoya [67] Othman [68]^a^ Rajan [69]^a^ Caudill [70]^a^

^a^Previously validated

#### Validated survey tools

Only three survey tools assessing knowledge, beliefs and attitudes of physicians toward the interaction met our eligibility criteria for a ‘validated tool’ [2, 22, 23]. Table 2 provides a full description of those tools. In summary:

- *Concepts measured* The first tool assessed the physicians’ attitudes towards pharmaceutical companies representatives (PCRs) [22]. The second assessed the perspectives towards the importance of different “sources of influence” [2]. The third assessed physicians’ attitudes toward interacting with PCRs and the perceptions of the effect of the value of a gift on physician’s judgment [23].
- *Development methods* All three validated survey tools were developed based on review of literature. The first tool was additionally developed using in-depth interviews of physicians and PCRs to develop a preliminary questionnaire [22]. The second [2] and the third [23] survey tools were developed by consulting with experts while developing the questionnaire. Pre-testing was conducted for the first [22] and second [2] tool.
- *Types of validity* Construct validity and factorial analysis were reported for the development of all three tools [2, 22, 23].

## Discussion

In summary, the purpose of this study was to systematically review the available tools to survey physicians about their interactions with pharmaceutical company and identify the validated ones. We identified one validated questionnaire assessing the nature and extent of the interaction, and three validated questionnaires assessing knowledge, beliefs and attitudes of physicians toward the interaction. None of these validated questionnaires were used in more than one survey. Four of 58 surveys on interactions used validated questionnaires.

The major strength of our study is the use of systematic review methodology (including comprehensive search, duplicate selection and data abstraction processes). Also, our systematic review is the first one to assess the validity of tools to survey physicians about their interactions with pharmaceutical companies. One potential limitation is our restriction to physicians in practice. Although one could argue that questionnaires designed to survey residents and students might be informative and used for physicians, we wanted our study to be more focused.

Unfortunately, the use of non-validated or poorly validated instruments is not uncommon in health survey research. Indeed, we have identified three methodological studies assessing the validity of tools in specific healthcare fields [28–30]. The first study included studies assessing the attitudes of healthcare students and professionals towards patients with physical disability [28]. This study identified 38 eligible surveys, nine of which used validated instruments, and only three of these fit our validity criteria [28]. The second methodological study focused on studies assessing knowledge, perceptions and practices of health care professionals towards alcoholic patients [29]. Out of 21 included studies, the numbers assessed for internal construct validity, external construct validity, and predictive validity, internal consistency, and reliability were 8, 15, 1, 7 and 0 respectively [29]. The third study focused on surveys of prevalence instruments in clinical and epidemiological research on waterpipe smoking [30]. Out of 38 identified surveys none one reported using a validated instrument [30]. The lack of consistent use of validated instruments could be a combination of substandard conduct and a substandard reporting of survey studies.

## Conclusion

Policy makers addressing the issue of physicians’ interaction with pharmaceutical company need to be aware of the low quality of supporting evidence due to the use of non-validated questionnaires (given the bias that could be introduced into the findings). One observation is that the questionnaires in the identified surveys measured different concepts (e.g., aspects of the interaction, “information” or the “gifts” aspect). This limits the capacity of comparing results across studies (e.g., across different countries, or in the same country over time).

Our findings highlight the importance of the use by investigators of commonly accepted and validated instruments. These investigators could use our findings to choose a validated questionnaire. Unfortunately the choices are limited, and those investigators may reasonably judge that none of the instruments address exactly their specific research question. Therefore there is a need for research to develop and validate such tools. Investigators also need to adhere to suggested guidelines for reporting survey studies that include recommendations for reporting the extent to which the validity and reliability of the instrument have been established [31]. It would also be ideal for journals to require authors of surveys to adhere to those guidelines.

## Appendix 1: Types of validity

1. Content Validity: refers to evaluation of the items of a measure to determine whether they are representative of the domain that the measure seeks to examine. It is based only on the judgment of experts regarding the content of the items.
2. Face Validity: assumes that when we look at the questions included in a measuring instrument, it appears to measure the concept that it intends to measure. It refers to how users of the instrument perceive it.
3. Criterion Validity: establishing a correlation between the measure and an external criterion (a gold standard).
4. Construct Validity: the degree of measurement of a theoretical concept, trait, or variable; the way in which our construct behaves or correlates with other related constructs. In other words, it represents a framework of hypothesis testing based on the knowledge of the underlying construct.

## Appendix 2: Search strategies

### Ovid MEDLINE(R) <1946 to August Week 4 2015>

1. Conflict of Interest.mp. or “Conflict of Interest”/ (9234)
2. Drug Industry/ (30016)
3. Gift Giving/ (1306)
4. detailman.mp. (4)
5. commercial information.mp. (30)
6. ((drug or pharma*) adj3 (industry or firm* or manufacture* or compan*)).mp. (40649)
7. physician*.mp. (423538)
8. doctor*.mp. (89855)
9. Physicians/ (65190)
10. primary care.mp. (72237)
11. or/1-6 (48354)
12. or/7-10 (522090)
13. 11 and 12 (5684)
14. 13 not (comment or editorial or letter).pt. (4745)

### Embase <1980 to 2015 Week 36>

1. Conflict of Interest.mp. or “Conflict of Interest”/ (9927)
2. Drug Industry/ (68082)
3. Gift Giving/ (962)
4. detailman.mp. (3)
5. commercial information.mp. (47)
6. ((drug or pharma*) adj3 (industry or firm* or manufacture* or compan*)).mp. [mp=title, abstract, heading word, drug trade name, original title, device manufacturer, drug manufacturer, device trade name, keyword] (97838)
7. physician*.mp. (519656)
8. doctor*.mp. (200550)
9. Physicians/ (163052)
10. primary care.mp. (102606)
11. or/1-6 (106455)
12. or/7-10 (708268)
13. 11 and 12 (8296)
14. 13 not (comment or editorial or letter).pt. (7157)

## Appendix 3: Details of the validated tools

Madhavan surveyed physicians in West Virginia about their attitudes “surrounding the ‘gift relationship’ between pharmaceutical companies and physicians”. The questionnaire developed for the purpose of this study consisted of three sections: the first included statements to which physicians would indicate how much they agree or disagree, the second aimed to collect demographic information and physicians’ practice information, and the third asked about the amount and value of “gifts” they recently received. Examples from the first section: “pharmaceutical companies give gifts to physicians to influence their prescribing”, “it is inappropriate to accept gifts from pharmaceutical companies” and “physician-patient relationship would be improved if the extent of the gift and receiving relationship between pharmaceutical companies and physicians was made public”.

McKinney was the oldest published study (1990) about attitudes of physicians and residents toward their “professional interaction with pharmaceutical sales representatives”. The participants were from Minnesota and Wisconsin. The questionnaire they developed had two parts, the first asked the physicians about the potential ethical compromise when receiving gifts from PCRs and the frequency of their contact with the PCRs; the second included statements assessing attitudes towards PCRs and the physicians reported the extent of their agreement with these statements. Some of these statements were “pharmaceutical representatives provide useful and accurate information about newly introduced drugs”, “pharmaceutical representatives should be banned from presentations at this institution”, “I would have the same degree of contact with pharmaceutical representatives whether or not promotional gifts were distributed” and “acceptance of promotional items from pharmaceutical representatives has no impact on my prescription behavior”.

Andaleeb [22] assessed the attitudes of physicians in Pennsylvania toward PCRs. They formulated the questionnaire’s items after reviewing the literature and gathering information from physicians and PCRs directly, and then conducted in-depth interviews to identify elements explaining physicians’ attitudes. The preliminary questionnaire was pretested on three physicians and then modified. The final questionnaire included four subgroups and each had a statement to which physicians would rate their agreement to. The subgroup “favor” included “pharmaceutical sales representatives are an asset to my practice”; “support” included “pharmaceutical sales representatives provide me with information that helps me practice better medicine”; “Style” included “I feel pharmaceutical sales representatives are always trying to manipulate me to prescribe their company’s brands”; and “peers” included “the medical community has a negative attitude toward detail persons”.

Fernandez [2] aimed to understand the “opinion of general practitioners on the importance and legitimacy of sources of influence on medical practice”. The physicians from two Spanish regions were asked to assess the legitimacy of different strategies and/or groups on influencing medical practice: “financial incentives, politicians of the health field, pharmaceutical industry, scientific organizations, academic institutions and professional associations”. Another task was to rate the importance of different change strategies to medical practice: “information provided by pharmaceutical companies’ visitors”, “attendance at training courses, reading articles and reports” and “existence of financial incentives”.

## Abbreviation

PCR: pharmaceutical company representatives
